# Genetic Variability in Seed Longevity and Germination Traits in a Tomato MAGIC Population in Contrasting Environments

**DOI:** 10.3390/plants12203632

**Published:** 2023-10-20

**Authors:** Elise Bizouerne, Joseph Ly Vu, Benoît Ly Vu, Isidore Diouf, Frédérique Bitton, Mathilde Causse, Jérôme Verdier, Julia Buitink, Olivier Leprince

**Affiliations:** 1 Institut Agro, INRAE, University Angers, IRHS, SFR QUASAV, 49000 Angers, France; elise.bizouerne@gmail.com (E.B.); joseph.ly-vu@inrae.fr (J.L.V.); benoit.lyvu@agrocampus-ouest.fr (B.L.V.); jerome.verdier@inrae.fr (J.V.); julia.buitink@inrae.fr (J.B.); 2 Génétique et Amélioration des Fruits et Légumes, Centre de Recherche PACA, INRAE, UR1052, CS60094, 84143 Avignon, Francefrederique.bitton@inrae.fr (F.B.); mathilde.causse@inrae.fr (M.C.)

**Keywords:** ageing, germination, heat stress, longevity, multiparental population, phenotypic plasticity, QTL, *Solanum lycopersicon*

## Abstract

The stable production of high vigorous seeds is pivotal to crop yield. Also, a high longevity is essential to avoid progressive loss of seed vigour during storage. Both seed traits are strongly influenced by the environment during seed development. Here, we investigated the impact of heat stress (HS) during fruit ripening on tomato seed lifespan during storage at moderate relative humidity, speed (t50) and homogeneity of germination, using a MAGIC population that was produced under optimal and HS conditions. A plasticity index was used to assess the extent of the impact of HS for each trait. HS reduced the average longevity and germination homogeneity by 50% within the parents and MAGIC population. However, there was a high genetic variability in the seed response to heat stress. A total of 39 QTLs were identified, including six longevity QTLs for seeds from control (3) and HS (3) conditions, and six plasticity QTLs for longevity, with only one overlapping with a longevity QTL under HS. Four out of the six longevity QTL co-located with t50 QTL, revealing hotspots for seed quality traits. Twenty-one QTLs with intervals below 3 cM were analyzed using previous transcriptome and gene network data to propose candidate genes for seed vigour and longevity traits.

## 1. Introduction

Seed vigour is an estimate of how successful a seed lot will establish seedlings under a wide range of environmental conditions. Therefore, it has a direct impact on crop yield [1]. Tomato seed vigour characteristics includes a high, rapid and synchronous germination, absence of dormancy, a uniform establishment of seedlings as well as the absence of abnormal seedlings [2,3,4]. Seed longevity is defined as the capacity to remain alive during dry storage, and is an essential trait to slow down the progressive loss of seed vigour over time during storage. Therefore, it is important to understand the genetic architecture that controls seed longevity and how it is linked to other seed vigour traits.

Genetic studies on seed longevity have succesfully led to the identification of many QTL and candidate genes in wild species including Arabidopsis, *Medicago truncatula*, field crops such wheat, barley, maize, rice, soybean, as well as vegetable crops such as lettuce and tomato (reviewed in [5,6]). In tomato, only few studies investigated the genetic architecture of longevity. In a study on 50 recombinant inbred lines (RIL) from a cross between *Solanum lycopersicum* cv. Moneymaker and *S. pimpinellifolium* identified 3 QTLs of longevity on chromosomes 2 and 6, assessed as germination after 15 days of storage at 40 °C and 85% RH [7]. A QTL for longevity on chromosome 2 colocated with a QTL for galactinol content, with in the interval a galactinol synthase (GolS) gene. Consistent with this, Arabidopsis mutants for GolS genes were affected in longevity, suggesting that galactinol content of mature dry seeds can be used as a biomarker for seed longevity. The same population was studied for seed vigour traits by Kazmi et al. [2], focussing on the speed of germination under different abiotic stresses. The longevity QTL on the top arm of chromosome 2 colocated with germination speed under salt, osmotic and oxidative stress.

A genetic link between longevity and other seed vigor traits has also been established in Arabidopsis using a recombinant inbred line (RIL) population derived from a cross between the accessions Landsberg erecta (Ler) and Shakdara (Sha) [8]. Using 85% RH and 40 °C and ambient laboratory conditions as storage conditions, a longevity QTL was identified that colocated with germination speed, and germination under salt stress, showing that this locus had pleiotropic effects [8]. When QTL analyses for seed longevity using six RIL populations were performed under storage at ambient conditions, the major seed longevity QTL colocated with seed dormancy QTL [9]. Deep dormancy was correlated with low longevity, suggesting that longevity and dormancy are genetically negatively correlated. *GAAS5*, a major longevity QTL was the same locus as *DELAY OF GERMINATION 1* (*DOG1)* and further analysis showed that the Cvi allele of *DOG1* was responsible for both higher seed dormancy and lower longevity [9]. The precise role of DOG1 in seed longevity remains to be elucidated [10]. In the tomato Moneymaker genotype, we, previously, found that the acquisition of longevity during seed maturation was concomittant with a decrease in dormancy [11]. Using a gene network analysis based on transcriptomes specific of developing embryo, endosperm and seed coat together with gene-trait correlations, we revealed a common gene module between the endosperm and embryo that contained longevity associated genes related to antioxidant, repair and protective mechanisms together with *SlDOG1–2*. The embryo-specific module contained homologues of *ABA-insensitive4 (ABI4)* gene whose role in seed longevity has recently been demonstrated in *Medicago truncatula* [12]. In tomato, the endosperm-specific module revealed diverse processes involved in genome stability and ABA/GA response genes [11]. However, it is still unclear whether these candidate genes can explain the natural variation that exists in longevity and other vigor traits such as dormancy or speed of germination.

Identifying polymorphisms controlling quantitative traits such as longevity remains a challenge for plant variety selection. Most studies on longevity QTL mapping in crops, including tomato relied on populations from two-parent crosses [6]. However, these populations only allow the analysis of alleles differing between two lines, and have a resolution limited to 10–30 cM, as the analysis mainly relies on recombination events occurring during meiosis in the F1 generation [13]. The previous longevity QTL based on *S. pimpinellifolium* (the most recent wild ancestor of the cultivated tomato) and a cultivated accession provided valuable genetic information but, because of undesirable linkage drag that comes often with wild progenitors, these QTLs might be difficult to use effectively in a breeding program [14]. Multiparental populations such as Multi-allelic Genetic InterCross (MAGIC) population are intermediate populations to two-parent and Genome-Wide Association Study (GWAS) populations, with more balanced allele frequencies than GWAS panels and more efficient recombination than two-parent populations. The use of a MAGIC population makes it possible to exploit intraspecific variation, to do QTL mapping but also to identify causal polymorphisms. The studied tomato MAGIC population was created from 8 parents selected to have a wide range of genetic diversity, with 4 parents bearing small fruits (i.e., Cervil, Criollo, Plovdiv24a and LA1420) and the other 4 parents bearing large fruits (i.e., Levovil, Stupicke Polni Rane, LA0147 and Ferum) [15]. The genomes of the 8 founding parents were sequenced and more than 4 million SNPs were identified compared to the reference tomato genome, out of which 1536 SNPs were selected to build a genetic map. The linkage map obtained with this population showed an increase of 87% in recombination frequencies compared to two-parent populations and the detection of QTL was made possible at the haplotype level [14,15]. Therefore, this material together with our tissue-specific seed maturation network [11] represent valuable resources to assess the genetic architecture of longevity.

Another important factor controlling longevity is the growing conditions during seed development (reviewed in [5,6]). Temperature is one of the most important determinants of seed dormancy and longevity as it affects the genetic programs leading to the acquisition of the different traits characterizing seed vigour traits [16,17]. In *Arabidopsis,* low temperatures during seed maturation lead to deep primary dormancy and decreased longevity compared to optimal conditions [17,18]. In barley, the longevity was also dependent on the location where seed lots were obtained [19]. In tomato, field conditions during seed production [20] and different supply of nitrate and phosphate during plant growth [21] impacted both the seed physical characteristics, germination and seedling emergence. With the prospects of global climate change, especially increasing temperatures, it is important to investigate whether interactions between heat stress during seed production and genotype influences seed longevity and seed vigour.

The aim of this work was to explore the genetic diversity of seed survival during storage at 35 °C and 75% RH and identify longevity QTLs using the MAGIC population that was cultivated in greenhouse conditions [14]. We also investigated the impact of heat stress during fruit ripening on seed longevity and speed of germination after one year of after-ripening A narrowed list of candidate genes was proposed by integrating the QTL results with the differentially expressed genes associated with seed maturation [11,22].

## 2. Results

### 2.1. Phenotypic Variation of the Germination and Longevity Is Explained by Genotype, Maternal Temperatures and GxE Interaction

**Figure 1:**
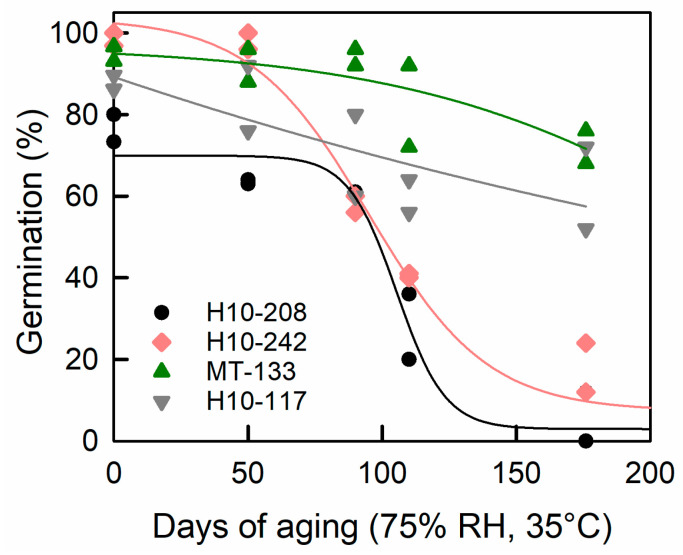
Survival curves of seeds from four accessions of the MAGIC population during storage. Seed lots obtained under control conditions were equilibrated at 75% RH for 7d, and thereafter stored in hermetically sealed pouches at 35 °C. Seeds were considered germinated when the radicle protruded the seed coat. Data represents two batches of 50 seeds.

The MAGIC population was cultivated in a greenhouse under control and heat stress conditions [14]. The daily mean/maximal temperatures were 21.2 °C/28.8 °C in control and 26.9 °C/34.4 °C in heat stress conditions. Longevity was determined after a defined time of storage, since not enough seeds were available for all accessions to establish full survival curves. A pilot experiment carried out on a restricted number of genotypes, indicated that 110 days of storage was the best time point to capture the genetic variation of seed longevity (Figure 1). To assess seed vigour, the following traits were phenotyped: germination (i.e., germination after 8 days on water at 20 °C in the dark), germination capacity (i.e., germination after dormancy release treatment using KNO_3_ and stratification, speed of germination (time to reach 50% germination, t50), germination homogeneity (time difference between 20 and 80% germination, t80t20).

**Figure 2:**
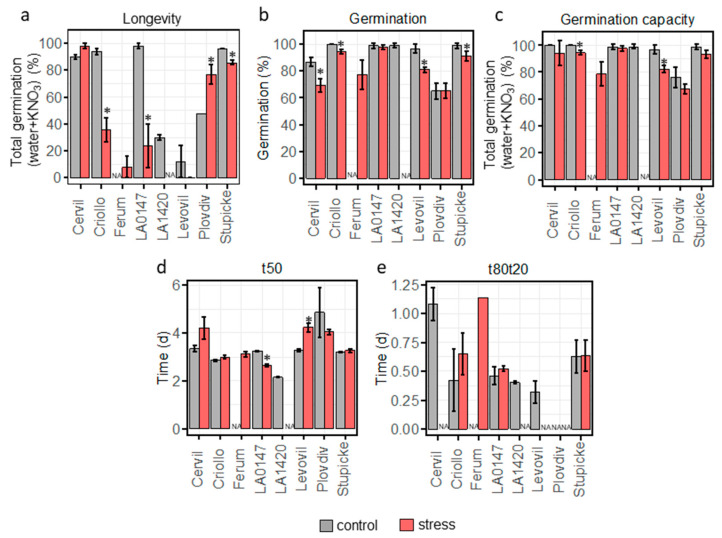
Seed vigour traits for the 8 MAGIC parental lines. (**a**) longevity (percentage of germination after 110d of storage at 35 °C and 75% RH), data are the mean of duplicates (with minimum and maximul values as error bars) of 50 seeds. (**b**) percentage of germination, (**c**) percentage of germination capacity, (**d**) germination speed (t50), (**e**) germination homogeneity (t80t20). Data are the mean of triplicates (±SD) of 30 seeds. Stars indicate significant difference between control and heat stress using a z-test (*p* < 0.05) for longevity and a *t*-test (*p* < 0.05) for germination, germination capacity, t50 and t80t20.

A large variation in longevity was found among the 8 parental lines. For control conditions, Cervil, Criollo, LA0147 and Stupicke showed the highest germination percentage after 110 days of storage whereas Levovil and LA1420 showed a poor longevity with respectively 11 and 30% of germination after storage. Plovdiv had intermediate storability with 48% of germination after storage (Figure 2a). Heat stress during fruit ripening significantly reduced by half the storability of Criollo and LA0147 and on the contrary increased Plovdiv seed longevity (Figure 2a), showing that the plasticity of longevity is genotype dependent. All parent lines produced in control conditions showed a final germination higher to 80% except Plovdiv for which only 65% of the seed batch germinated (Figure 2b). Heat stress had a significant negative impact on germination in Cervil, Criollo, Levovil and Stupicke (Figure 2b). Germination capacity, corresponding to germination after KNO_3_ and stratification treatment in order to release residual primary dormancy, was the lowest in Plovdiv with 76% germination while the other genotypes showed more than 95% germination (Figure 2c). Heat stress negatively influenced germination capacity of Criollo and Levovil (Figure 2c). The faster germination speed was observed in LA1420 with a t50 of 2.2 days while Plovdiv had the slowest germination speed with 4.8 days (Figure 2d). Heat stress respectively reduced and increased t50 in LA0147 and Levovil (Figure 2d). Germination homogeneity (t80t20) varied from 0.3 days for Levovil to 1.1 days for Cervil (Figure 2d). No t80t20 data are available for Plovdiv and for Cervil, LA1420, Levovil under stress conditions because the seed lot did not reach 80% of germination. For Criollo, LA0147 and Stupicke, heat stress had no effect on germination homogeneity (Figure 2e).

**Figure 3:**
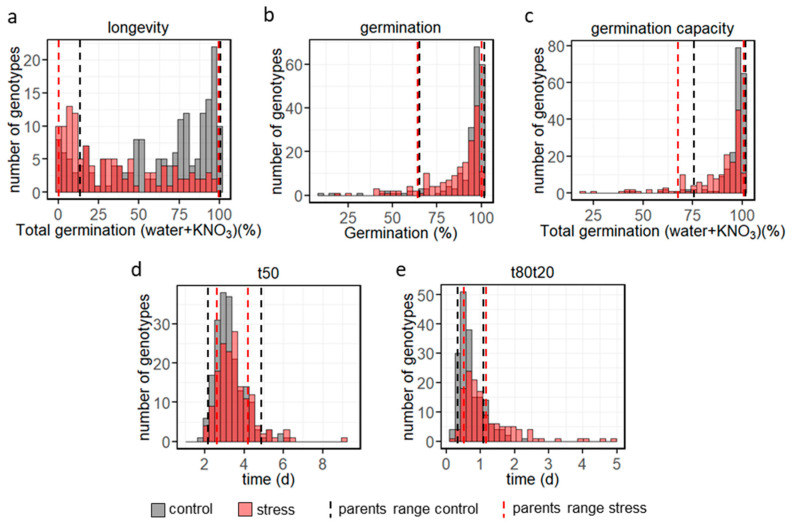
Frequency distribution in the MAGIC population for (**a**) longevity (germination after 110d of storage), (**b**) germination, (**c**) germination capacity, (**d**) germination speed (t50) and (**e**) germination homogeneity (t80t20), under control (grey) and heat stress (light red) conditions. Vertical dashed lines represent the MAGIC parental lines range under control (black) and heat stress (red) conditions.

The seed traits were assessed in seeds from 187 and 121 lines for longevity from control and heat stress conditions, respectively, and in 204 and 164 lines for germination, germination capacity, t50 and t80t20 on seeds obtained from control and heat stress conditions, respectively. Within the population, longevity was the trait most impacted by heat stress (Table 1). Average longevity across the MAGIC population was reduced from 62% of germination after storage in control seeds to 32% in seeds from plants grown under heat stress. In stressed seeds, the majority of genotypes showed less than 40% germination after storage (Figure 3a). Heat stress also decreased the mean homogeneity of germination from 0.7 to 1.2 days (Figure 3e, Table 1). A high genetic variability was observed for longevity with a distribution of germination percentages after 110 days of storage ranging from 0 to 100% across genotypes (Figure 3a). For the control condition, frequency distribution of germination and germination capacity percentages showed that most genotypes germinated above 80% (Figure 3b,c). These high germination percentages and the absence of a large difference with the percentage of germination capacity highlighted the absence of primary dormancy in most of the MAGIC lines, likely because testing was performed 20 months after harvest. For germination speed, a high genetic variability was observed with t50 ranging from 1.9 to 6.3 days for control growth conditions (Figure 3d). Frequency distribution for germination homogeneity also showed a high genetic variability with t80t20 ranging from 0.2 to 2.3 days in control condition (Figure 3e). For all traits the highest values in the MAGIC lines always exceeded the maximum parental values (Figure 3, Table 1). For control environment, estimates of broad sense heritability for the five traits were high, ranging from 0.72 for t80t20 to 0.95 for t50 (Table 1). Heat stress had no effect on heritability values for germination capacity and longevity but slightly decreased those for germination, t50 and t80t20.

A two-way ANOVA model was used to investigate how phenotypic variation was attributed to the genotype, the environment and their interaction (GxE) by partitioning the total sum of s–quares. Consistently with the high heritability values, a large part of the phenotypic variation was linked to the genotype ranging from 52 to 70% of the total sum of squares (Table 2). A significant effect of the environment was found for all traits with the proportion of total sum of squares ranging from 1 to 13% respectively for t50 and longevity. These low values might be due to the high number of genotypes compared to the low number of environments (i.e., control vs. heat) tested in our study. All traits showed a significant GxE interaction ranging from 19 to 27% of the total sum of squares (Table 2).

**Figure 4:**
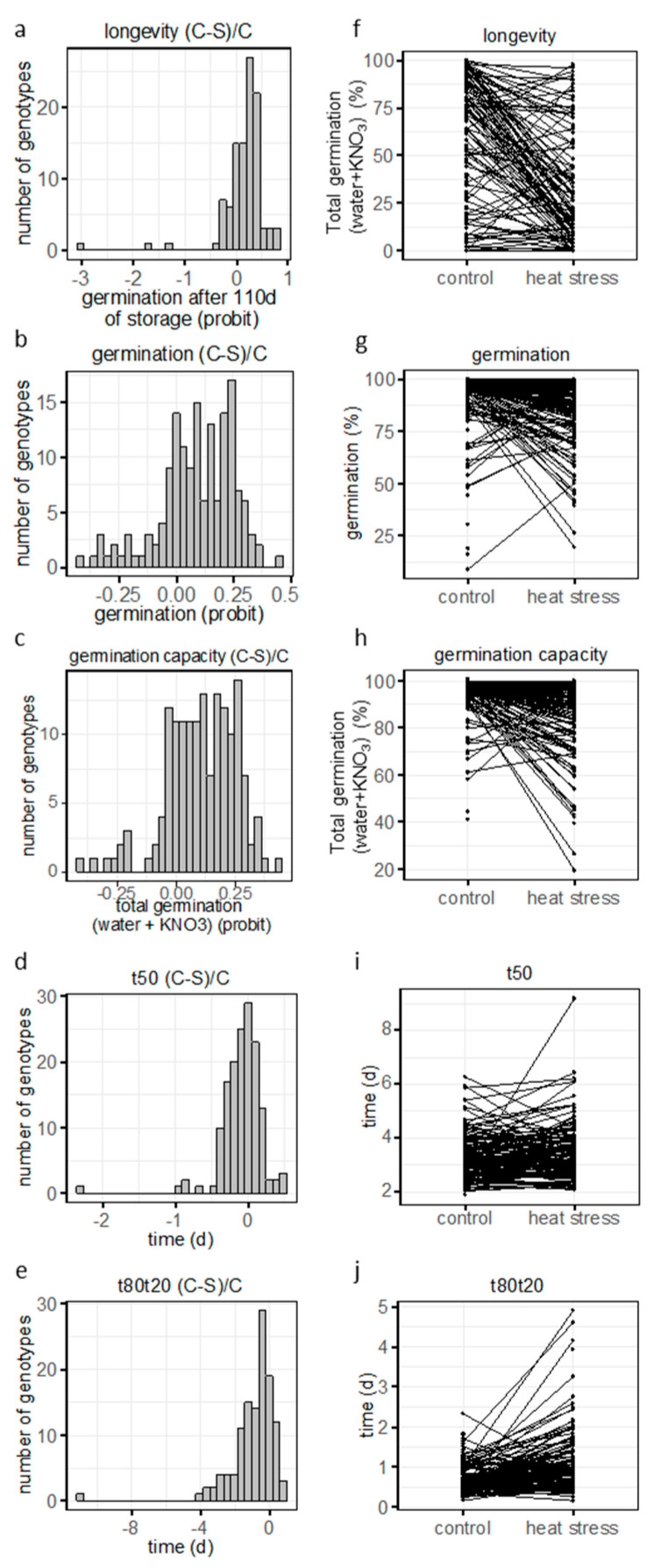
Frequency distribution of phenotypic plasticity (**a**–**e**) and reaction norm plot (**f**–**j**) for longevity (germination after 110d of storage) (**a**,**f**), germination (**b**,**g**), germination capacity (**c**,**h**), t50 (**d**,**i**) and t80t20 (**e**,**j**). Phenotypic plasticity was calculated as [(C-S)/C]. Percentage of germination before or after storage or cold treatment were transformed into probit.

Considering the significant GxE effects (Table 2), we addressed whether and how plasticity of germination traits and longevity due to growth conditions varied across the MAGIC population. For this purpose, pairwise differences between control (C) and heat stress (S) conditions for each genotype were calculated for values expressed as differences between control and stress relative to control [(C-S)/C] (Figure 4). Data are shown as histograms reflecting the frequency distribution with 0 indicating no plasticity (Figure 4a–e) and as reaction norm plots (Figure 4f–j)). For longevity, the frequency distribution showed a broad distribution in plasticity values (Figure 4a). The reaction norm plot showed that 79 genotypes exhibited a decreased longevity when seeds were produced under heat had positive phenotypic plasticity values (Figure 4f). Most genotypes with more than 75% germination after storage in control conditions had less than 25% germination in heat stress condition. However, 15 genotypes showed the opposite trend, i.e., an increased longevity when seeds were produced under heat stress (Figure 4a,f). Frequency distribution of the plasticity of germination and germination capacity showed that the majority of genotypes exhibited a distribution towards positive values (Figure 4b,c). It is noteworthy that 14 and 6 genotypes for germination and germination capacity, respectively, exhibited an increase in germination when seeds were grown under heat stress (Figure 4g,h). We suspect that high temperatures during seed development might have made it easier for these genotypes to undergo after-ripening during storage compared to seeds produced under control conditions [23]. For t50, frequency distribution of plasticity showed that the majority of the genotypes were centred around 0. Twenty genotypes had a higher t50 in control condition than in heat stress (Figure 4d,i) whereas the opposite trend was found for 53 genotypes exhibiting a lower t50 in control compared to heat stress (Figure 4d,i). For t80t20, phenotypic plasticity values were mostly distributed towards negative values, showing that heat stress broadened the speed of germination of offsprings, resulting in a decrease in the homogeneity of germination (Figure 4e,j).

### 2.2. QTL Identification for Seed Longevity and Germination Traits

**Figure 5:**
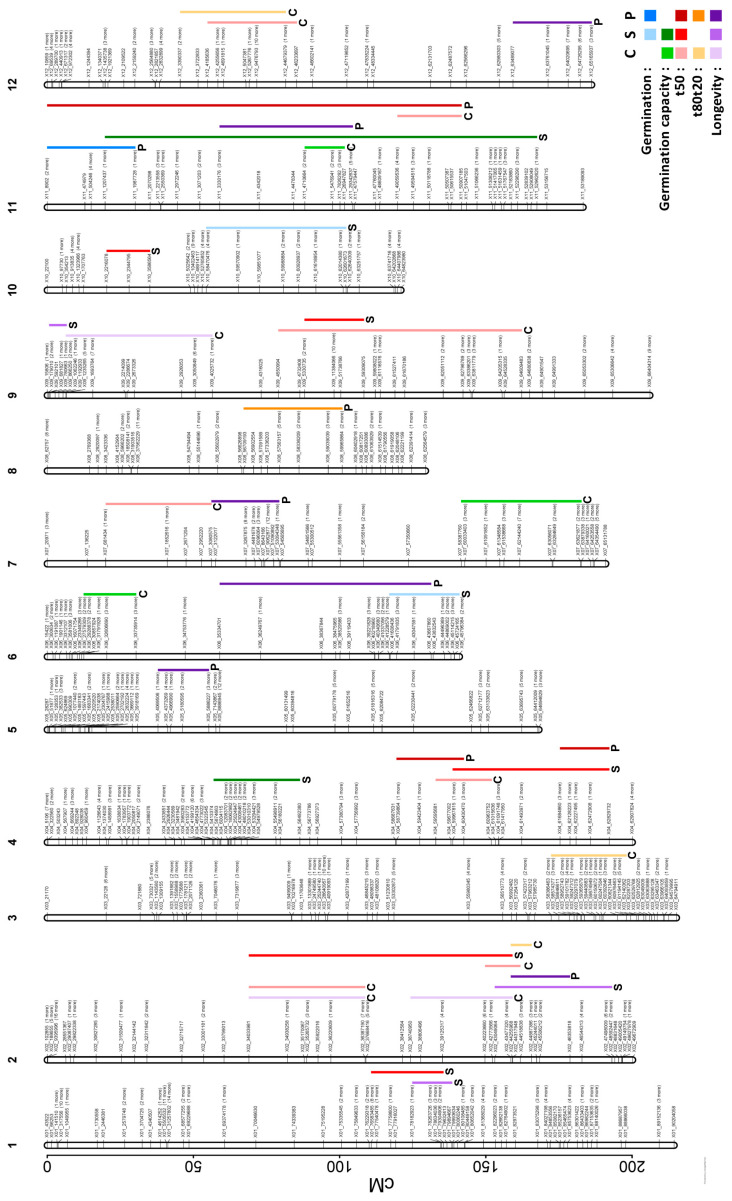
Detected QTL position on the genetic map depicting the 12 chromosomes. Germination (blue), germination capacity (green), t50 (red), t80t20 (orange), longevity (purple). Control condition (C, light colour), heat stress condition (S, bright colour) and plasticity (P, dark colour).

To identify potential loci regulating seed longevity and germination characteristics, QTL mapping was performed with phenotype data obtained on seeds from both control and heat stress conditions. For both growth conditions, a total of 27 QTL were detected for the five studied traits with confidence intervals ranging from 5.9 to 90.5 cM (0.64 to 60.1 Mb) and LOD values from 4.15 to 6.40 (Figure 5, Table 3). Six QTLs were found for longevity (three for control and three for heat stress), with one stable QTL on chromosome 2. For the seed vigour traits, two QTLs were detected for germination, four for germination capacity, 12 for t50 (seven for control and five for heat stress), and three for t80t20 from seeds grown under control conditions (Table 3). Three stable QTL for t50 were found on chromosomes 2, 4 and 9 (Figure 5).

For the six longevity QTLs, four QTLs co-located with t50 on chromosomes 1 and 2, with the one located at the lower part of the chromosome 2 also co-locating with a QTL for t80t20, showing that this region is a hotspot for physiological traits. Other co-locations were found among the QTLs for the different germination traits. One QTL for germination and germinative capacity co-located on chromosome 11, which can be explained by the high correlation coefficient (PCC = 1) between the two traits (Figure S1). The QTLs for t50 and t80t20 that were obtained from seeds grown under control conditions shared the same intervals on chromosomes 2 and 12.

### 2.3. QTLs of Phenotypic Plasticity of Longevity and Seed Germination (pQTL)

**Figure 6:**
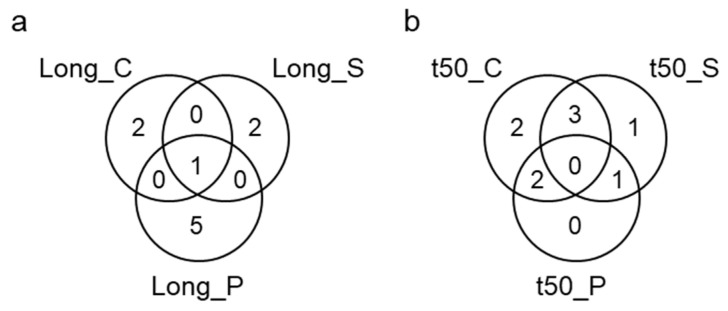
Venn diagram depicting the overlap in QTL intervals between longevity (**a**) and t50 (**b**) QTLs.

To investigate genetic variation in phenotypic plasticity, pQTLs were determined on the plasticity values presented in Figure 4. A total of 10 pQTLs were identified, with six for longevity, two for t50, and one for germination and t80t20 (Figure 5, Table 3). Five of the six pQTL of longevity were different from those identified for longevity QTLs under control or stress conditions, indicating that genetic factors explaining this plasticity are different from the genes that regulate the longevity directly (Figure 6). The only pQTL of longevity that co-located with a QTL of longevity obtained under stress on chromosome 2 also co-located with a QTL for t50 and t80t20 (Figure 5 and Figure 6). The other longevity pQTLs were found on chromosomes 5, 6 (sharing the same interval with germination QTL obtained under stress), 7, 11 (sharing the same interval with a germinative capacity QTL), and 12 (Table 3). For the speed of germination, the three pQTLs were all co-locating with either t50 QTLs obtained from seeds grown under control or stress conditions (Figure 6).

### 2.4. Identification of Candidate Genes under the QTLs

Following QTL identification, the genes present in the confidence intervals of the 21 QTL mapped in a region smaller than 3Mb were assessed (Table 3 and Table 4). The number of candidate genes under these 21 QTLs (ranging from 88 to 332) was narrowed down by contrasting the allelic effects of the parental lines (Table S1). This filter resulted in number of candidate genes ranging from 10 for a t50 QTL on chromosome 4 to 277 genes for the t50 QTL (X04_59595681) on chromosome 2 (Table 4, column ‘filtered nb CG’). A second filter was based on the hypothesis that genes that determine the QTLs are expected to be expressed in seeds. Using the gene expression profiles obtained during development of Moneymaker seeds from [11], we retained only those genes whose transcripts were ≥1 CPM in at least one developmental stage in one of the three seed tissues. After this filter, the number of retained candidate genes ranged from 6 for a t50 QTL on chromosome 4 to 230 genes for a t80t20 QTL on chromosome 3 (Table 4, column ‘seed expr CG’) (Table 4).

Next, for each QTL, the filtered candidate gene list was further investigated to identify the most likely candidate genes that could explain the QTL using knowledge from the literature and results produced in Bizouerne et al. [11]: (1) presence of the genes in modules that were associated with the release of longevity in both embryo and endosperm (ME2), in the endosperm (ME4) and in the embryo (ME7), (2) whether gene transcripts correlated with seed traits and 3) whether genes were seed and/or tissue preferentially expressed (Table S2).

#### 2.4.1. Candidate Genes under QTL of Longevity

Three longevity QTLs were detected on seeds grown under control conditions (Table 3). One QTL co-located with a t50 QTL obtained under control conditions on chromosome 2, with a total of 182 filtered genes in common between both intervals. Five DOG-like genes are found in this region, including two truncated proteins containing the DOG domain (Solyc02g072550.1.1 and Solyc02g072560.1.1, Table S2). Transcript levels of Solyc02g072550.1.1 strongly correlate with the acquisition of longevity and dormancy release and the gene is part of the ME2 gene module [11]. One other DOG1/TGA like domain protein is Solyc02g073580.1.1, with homology to DOG1-like 4. Analysis of the allelic variation between Cervil and LA0147 identified 88 polymorphisms with two of them leading to a non-synonymous modification in exon 1 that might explain variation in t50 (Table S3). Two additional genes (Solyc02g072570.2.1 and Solyc02g073570.1.1) are homologues of At4g18690, annotated as a DOG protein. In tomato, both genes are seed specific, with Solyc02g072570.2.1 located in the endosperm-specific gene cluster ME4 [11]. Among the 48 polymorphisms identified for Solyc02g072570.2.1, three of them led to a non-synonymous modification in exon 1 (Table S3). Two other genes that are known for their role in germination/protection are also present in this interval: Solyc02g070430.3.1, a homologue of *GIBBERELLIN 2-OXIDASE 1* (*GA2OX1)* and Solyc02g072550.1, a homologue of GASSHO1 (GSO1, At4g20140), a receptor kinase for which the Arabidopsis mutant exhibits defects in the embryonic cuticle integrity [24] and a slight decrease in germination after similar storage conditions as this study [25].

The second longevity QTL obtained for seeds grown under control conditions was part of a hotspot of seed vigour QTL, coinciding with a QTL for longevity (obtained under stress), t50 (control), t8020 (control), and the plasticity of longevity (Table 3). The number of genes is 667 in the whole interval containing all the QTLs and 35 in the interval overlapping between all QTL. Several genes are interesting candidates: Solyc02g087310.3.1 encodes a E2F transcription factor-like (At3g01330) that is part of the DREAM complex involved in the repression of growth in response to DNA damage [26]. Solyc02g087290.3.1 encodes a α-mannosidase that is present in ME2, a gene expression module characterizing late seed maturation [11]. Solyc02g086870.4 encodes a β-subunit of a farnesyl-transferase homologous to ENHANCED RESPONSE TO ABA 1 (ERA1, At5g40280). *era1* mutants are hypersensitive to ABA during germination [27] and exhibit a permeable cuticle [28].

The third longevity QTL detected from seeds produced under control conditions was located on chromosome 9. After filtration, 226 genes remained, with 181 being expressed during seed development. The tail of this QTL interval overlapped with a longevity QTL obtained under heat stress conditions. Only one gene overlapped between both QTLs: Solyc09g007670.3, a transducin/WD4 repeat-like superfamily protein whose role is unknown (Table S3). Amongst the other putative candidate genes, a homologue of *ETHYLENE INSENSITIVE 2* (*EIN2,* Solyc09g007870.3.1) was retained because its transcript profile was highly correlated with the acquisition of longevity in both the embryo and endosperm [11]. We identified 15 polymorphisms in *SlEIN2*, including two upstream SNPs (Table S3). We also identified two peptidylprolyl isomerases (Solyc09g008650.3.1, Solyc09g008410.3.1), implicated in protein folding in relation to damage after stress. Another putative candidate gene is Solyc09g010670.4 a homologue of DPA (At5g02470) which is also part of the DREAM complex mentioned above [26]

Three longevity QTLs were obtained on seeds collected from plants submitted to heat stress conditions. The QTL on chromosome 1 was found in the same interval as the QTL of t50 obtained under heat stress (Table 3). A total of 67 genes was in the same interval for both QTL. Amongst them, we found *PYL1* (Solyc01g095700.3.1), an ABA receptor that was present in the gene module ME2. On the second QTL that was partially co-locating with the QTL of longevity obtained under control conditions on chromosome 2 (see hotspot described above), we identified a homologue of DNA ligase 6 (Solyc02g091120.4.1). Using the allelic filter, 11 polymorphisms were detected in this gene, including three upstream SNPs and an indel (Table S3). The third heat stress QTL for longevity was detected on chromosome 9. Amongst the 30 genes that remained in the interval after allelic filtering, only one gene was preferentially expressed in seeds compared to other tissues: Solyc09g005970.1, an ABC transporter G family member. The Arabidopsis homologue *ATP-BINDING CASSETTE G2* (*ABCG2*, At2g37360) is for the synthesis of an effective suberin barrier in roots and seed coats. Seed coats of *abcg2 abcg6 abcg20* triple mutant plants had increased permeability to tetrazolium red and decreased suberin content [29].

#### 2.4.2. Candidate Genes under QTL of the Germination Traits

Besides the QTLs of the different germination parameters that co-locate with QTLs of longevity, a few QTLs were specific to germination. On chromosome 4, a region was identified containing a t50 QTL from control and heat stress conditions as well as a pQTL for t50 (Figure 4, Table 3). Under the heat stress QTLs of t50 on chromosome 4, we identified an endosperm specific ABA 8′hydroxylase (Solyc04g078900.3.1), involved in ABA degradation. Below the QTL of t50_S on chromosome 10 we identified Solyc10g008300.3, a mannan endo-1,4-β-mannosidase.

For t80t20, 306 candidate genes were identified within 2 QTLs on chromosome 2 and 3 (Table 4). Among the 230 genes in the QTL on chromosome 3, we identified a homologue of *TOPLESS* (*TPL*, Solyc03g117360.4.1) that is present in ME2. Four additional homologues of *TPL* were also that found that are included in the gene expression network (Table S2). No non-synonymous mutations were identified in the different *TOPLESS* genes (Table S3). Two genes that are present in the endosperm specific module ME7 were also found: an *EXTENSIN* (Solyc03g117190.1.1) and an *EXPANSIN* (Solyc03g115890.3.1).

#### 2.4.3. Plasticity of Longevity

To narrow down the list of candidate genes under the pQTLs of longevity, our tomato gene expression network that was established under a single (control condition) may not be suitable since it does not reflect the response of the developing seed to heat. Instead, we used a transcriptome data set of endosperm and embryo isolated from mature seeds for which the fruit ripening occurred at 23 °C/20 °C and 32 °C/26 °C [22]. Briefly, the heat stress resulted respectively in 1919 and 1267 differentially expressed genes (DEG) in the embryo and endosperm, respectively. For the pQTL on chromosome 5, allelic filtration retained 149 genes. Comparison with the transcriptome data on heat-induced genes [22], 6 genes were deregulated by the heat during fruit ripening (Solyc05g012060.4.1, Solyc05g010720.4.1, Solyc05g012070.3.1, Solyc05g012650.4.1, Solyc05g010240.4.1, Solyc05g010590.4.1, Table S2). The transcripts of an *ENOYL-CoAΔISOMERASE 1* (Solyc05g010720.4.1), a peroxisomal enzyme involved in lipid degradation were specific to the endosperm and negatively correlated with the acquisition of longevity. Consistent with a heat stress response, a gene encoding a member of chloroplast chaperonin complex (*CHAPERONIN-6-β4*, Solyc05g010240.4.1) was also found. Developing seeds of the *cpn60α2* mutant, another member of the chaperonin complex, were found to be temperature-sensitive [30]. The longevity pQTL on chromosome 12 contained 239 genes after allelic filtration, out of which 27 were deregulated by heat (Table S2). One of these genes is Solyc12g100270.2.1, encoding a member of the fatty acid hydroxylase superfamily whose Arabidopsis homologue (*CER1*) is involved in wax biosynthesis and cuticle development.

On the longevity pQTL on chromosome 2 co-locating with a longevity QTL obtained under stress condition, we retained candidate genes showing pleiotropic effects among the 28 DEGs present in the transcriptome. These include a Ninja-family protein AFP3 (Solyc02g088910.3.1) whose Arabidopsis homologue is *ABI5 BINDING PROTEIN 3* (*AFP3*). AFP proteins regulate the activity of ABI5, a hub gene controlling seed maturation, longevity and germination [10,31]. We identified 17 polymorphisms in *SlABF3* using the filter of the P50_stress QTL, including an upstream indel (Table S3). Several other candidate genes are associated with cell walls (e.g., WRKY transcription factor 3, Solyc02g088340.4.1; *ENDO-1,3(4)-β-GLUCANASE* 1, Solyc02g089730.1.1; COBRA-like protein, Solyc02g089120.4.1; hexosyltransferase, Solyc02g087350.3.1 whose Arabidopsis homologue is *GALACTINOL SYNTHASE 8*, At3g28340).

## 3. Discussion

Adverse growing conditions and particularly temperature imposed on the mother plant is known to impact the acquisition of seed vigour traits and longevity via poorly understood regulatory mechanisms. In this study, we characterized tomato seeds from a MAGIC population grown under control and heat stress conditions. We found that the temperature perceived by the mother plant and/or the developing seed impacts tomato seed longevity together with the speed and homogeneity of germination that is dependent on the genotype. These traits were characterized by high heritability at both optimal and high temperatures. The characterization of the MAGIC population led to the identification of 39 QTL associated with seed vigour and longevity. Poor longevity and delayed germination increase the odds of obtaining poor seedling emergence for transplant production in commercial settings [2]. In contrast to RIL obtained from biparental cross, the MAGIC population was derived from the cross of cherry and large-fruited parental lines already developed by breeders [15]. Altogether, this work provides efficient resources for variety selection under heat condition. It would be interesting to test other available Solanaceae MAGIC populations such as that from the intercrossing of seven cultivated eggplant (*Solanum melongena*) and one wild relative (*S. incanum*) parents [32].

Our QTL analysis extends a previous genetic analysis on yield components, phenology and fruit quality using the same genetic material [14]. These authors identified 51 heat-tolerant genotypes that could be used as breeding materials. The seeds used here were obtained from the same plants analyzed by Bineau et al. [14]. The longevity and speed of germination in optimal and heat conditions and the plasticity of these traits in these heat-tolerant genotypes were not significantly different from the rest of the genotypes of the MAGIC population. This suggests that the genetic architecture underlying the heat tolerance in vegetative and fruit tissues is not identical to that of the offsprings. Therefore, whether the maternal regulatory mechanisms provide to the progeny a phenotypic adaptation to local environmental conditions as described for dormancy [16,33] deserves further attention. Furthermore, the differences in the genetic architecture between seed and vegetative tissues poses real challenges in breeding for heat tolerance since yield should not be selected at the expenses of seed vigour.

### 3.1. The Polygenic Nature of Seed Longevity in Tomato

Overall, heat stress strongly decreased seed longevity with the MAGIC population. On average, germination percentage after 110 days of storage was 2-fold lower for seeds produced under heat stress than under control condition (Table 1). A similar negative effect of heat stress on seed longevity have been reported in *M. truncatula* and rice [5]. It is unlikely that such decrease is due to a deleterious effect of heat on the development of seeds resulting in defects in maturation. Firstly, we observed a wide range of survival after storage ranging from 0% to 100% within the heat-tolerant genotypes that had a similar flowering time. Secondly, only two longevity QTL identified were specific to the growing conditions whereas five plasticity QTL were found. This reinforces the idea that longevity, like dormancy, is a plastic trait. There are several genetic origins explaining the plasticity of longevity (reviewed in [14,34]). The observation that four out of the five pQTLs were found in different chromosome regions than QTLs of each growing condition (Figure 6) strongly suggests that longevity is regulated by a genetic regulatory network governing plasticity rather than an allelic sensitivity due to different environmental conditions. This is consistent with previous work on the plasticity of fruit quality and yield in tomato [14,34].

### 3.2. Candidate Genes under the Longevity QTL Reveal Protective Mechanisms Associated with Galactinol Synthesis, DNA Repair, Lipid Polyester Barrier Together with Ethylene and ABA Signaling

Like previous studies on other species, we identified a large genetic variation in tomato seed longevity. Using a RIL population from a cross between Moneymaker and *S. pimpinellifolium,* 3 longevity QTL were identified: two on chromosome 2 and one on chromosome 6 [7]. In our study, we also identified a QTL for seed longevity on chromosome 2. However, the galactinol synthase identified within the QTL on chromosome 2 by De Souza Vidigal et al. [7] was only 450 kb from our longevity QTL. We also found a galactinol synthase 8 on chromosome 2 under a pQTL of longevity. Galactinol synthases are responsible for the production of oligosaccharides of the raffinose family. Although mutants defective in the accumulation of these sugars exhibit a decreased longevity [7], their precise function is not yet understood. Differences of QTL of longevity between this work and that of de Souza Vidigal [7] could also be attributed to the different storage conditions. In their study, seeds were stored at 40 °C and 85% RH, which is much more deleterious than our storage conditions. The use of various seed ageing conditions makes it difficult to compare the molecular responses to seed ageing across studies, even within a species. In oilseed rape, almost all longevity QTL were dependent on the storage conditions [35]. Altogether, this suggests different mechanisms of protection against deterioration that depend on the storage conditions.

Our QTL analysis identified several genes that confer a protective barrier to the seed, thereby isolating the embryo from the seed external environment [25,36,37]. Here several candidate genes under different QTL were identified as playing a role in providing an impermeable barrier to the embryo such as *SlGASSHO1* on chromosome 2 that is involved in cutin integrity; a homologue of *ABCG2* on chromosome 9 involved in suberin formation and a homologue of *ECERIFERUM 1* on chromosome 12 involved in wax and cutin synthesis. In Arabidopsis seeds, the presence of cutin in the testa prevents water or oxygen from entering, thereby contributing to a better seed conservation [36]. In the *aberrant testa shape* (*ats*) mutant that is affected in the structure of the teguments, seeds show a strong reduction of longevity with only 20% germination compared to 95% for the wild type after 45 months of dry storage at room temperature [38]. The longevity of Arabidopsis *transparent testa (tt)* mutant seeds exhibiting modified testa flavonoid composition was reduced after long-term ambient storage [8,38]. Consistent with this, *TT5* (Solyc05g010320.4.1) encoding a chalcone-flavonone isomerase was found in the pQTL on chromosome 5 (Table S2).

Maintaining DNA integrity during seed storage is also important for seed longevity and several genes regulating DNA repair during germination have been demonstrated to be involved in Arabidopsis seed longevity such as DNA ligase and *ATM*, *ATR* and *SOG1*, which are genes regulating speed of germination in response to DNA damage [39]. A delay in germination is thought to allow for repair during imbibition before radicle emergence. A look at genes involved in DNA repair underlying longevity QTL identified the following candidates: on chromosome 2 an exonuclease 1 (Solyc02g071530.2.1 and a homologue of DNA ligase 6 (Solyc02g091120.4.1) whose Arabidopis mutant exhibits reduced longevity (references in [39]); and on chromosome 5 a DNA glycosylase (Solyc05g010800.3.1). Two additional candidate genes found on chromosome 2 and 9 are two different members of the DREAM complex, a master coordinator of cell cycle dependent gene expression and all DNA repair systems [26], and might represent an additional layer of regulation worthy of further investigation.

A regulatory gene involved in hormonal signalling that might be an interesting candidate gene for the longevity QTL on chromosome 9 is *SlEIN2,* a gene involved in ethylene signalling pathway. Several lines of evidence suggest a role of ethylene in seed longevity. In rapeseed, exogenous application of ethylene accelerated seed germination of aged seeds stored at 74% RH and 30 °C [40]. In tomato, after storage at 78% RH and 45 °C, germination of *never ripe* (*nr*) mutant seeds was reduced compared to wild type seeds [41]. Nr is an ethylene receptor and the dominant *nr* plants are insensitive to ethylene. How ethylene would control longevity remains to be determined. Recently EIN2 was found to fine-tune ABA responses during seed germination modulates ABA responses by indirectly targeting ABI5, independently of the canonical ethylene pathway [42]. Whether this regulatory process is associated with longevity in tomato remains to be investigated.

Following up on the putative role of EIN2 in ABA signalling, mutant seeds deficient or insensitive to ABA showed poor seed longevity [8]. For example, MtABI5 was found to regulate seed longevity in *M. truncatula* as *abi5* seeds showed a decreased in seed longevity compared to wild type [31]. This study revealed in the pQTL on chromosome 2 a homologue of AFP3, a negative regulator of ABA response that binds to ABI5 and regulates its transcriptional activity [43]. Functional validation of this gene would be interesting to test whether the fine tuning of ABA response might regulate the plasticity of longevity under heat.

### 3.3. QTL and Candidate Genes Involved in Seed Vigour

Several QTLs for the different germination traits were identified in our study (Figure 4). In a previous study using the cross between *S. lycopersicum* and *S. pimpinellifolium,* 4 QTLs for germination uniformity were identified on chromosome 3, 4, 7 and 8 [2]. According to their physical position on SL2.4, the QTL on chromosome 8 colocated with our t80t20 pQTL. Environmental conditions during seed multiplication differed largely between these studies. Therefore, this QTL might be responsible for the control of germination homogeneity according to the culture environment and highlights an interesting genome region to improve germination homogeneity.

We retained 5 homologues of *TOPLESS* (TPL) as potential candidate genes controlling germination homogeneity on chromosome 3 (Table S2). This protein is a transcriptional repressor of seed genes and a central player of primary dormancy in Arabidopsis. TPL interacts with AFP2 and AFP3 to form a transcriptional co-repressor complex controlling the expression of ABI5, also involved in dormancy and longevity. It also interacts with the DOG1-AHG1 complex that regulates dormancy via the regulation of ABI5 [44]. It remains to be investigated whether the intergenic or intronic polymorphisms affect *TOPLESS* expression.

As one of the candidate genes for t50, we retained two homologues of Sl*DOGL1* on chromosome 2, collocating with longevity in control conditions. These are interesting candidate genes owing its pivotal role in seed dormancy and longevity [9,10,23]. In Arabidopsis, DOG1 integrates the maternal temperature via ABA, GA and ethylene signalling pathways to control dormancy [23]. DOG1 affects the expression of hundreds of genes including *LATE EMBRYOGENESIS ABUNDANT* and *HEAT SHOCK PROTEIN* genes that provide protection during dry storage [10].

The role of DOGL4-1 is unclear. In Arabidopsis, seeds of the loss of function mutants *dogl4* had a lower germination and a slightly higher dormancy than wild-type suggesting that DOGL4 is a negative regulator of seed dormancy unlike DOG1 [45]. But these phenotypes were not confirmed in another study [46]. In addition to a low germination *dogl4-1* seeds showed a higher ABA sensitivity. This suggests that SlDOGL4 could negatively regulate ABA response. On the same chromosome region, we also noticed Sl*DOG1.* Among the 48 polymorphisms identified for this gene, three of them led to a non-synonymous modification in exon 1. The presence of *DOG1* and *DOGL4* on the QTL of germination speed concurs with the hypothesis that variation in t50 could be related to residual dormancy in some of the lines of our MAGIC population.

## 4. Materials and Methods

### 4.1. Plant Material

Up to 204 genotypes of the MAGIC population (described in [15]) were cultivated in 2017 in the greenhouse in Avignon, France under optimal conditions and heat stress conditions using the growth conditions and experimental design described in Bineau et al. [14]. Briefly, optimal conditions corresponded to a timely sowing whereas the heat stress conditions were obtained after sowing seeds two months later. The daily mean/maximal temperatures were about 21.2 °C/28.8 °C in control and 26.9 °C/34.4 °C in heat stress conditions, which corresponded to temperatures exceeding by about 6 °C the critical condition [14]). Plants grown under heat stress were not limited by irrigation and fertilization to avoid confounding effects with drought and nutrients, which is known to affect seed quality in tomato [47]. Mature red fruits were harvested around 3rd truss in early July for control and two weeks later for heat stress conditions. Seeds were extracted from at least seven fruits by incubating the locular tissues for 1h under gentle shake at room temperature in 100 mL of a 2.2% (*v*/*v*) HCl solution containing 40 mg of pectolytic enzymes (Lafazym CL^®^, Laffort, France). After extensive washing in water to remove remnants of fruit tissues, seeds were blotted dry on filter paper and rapidly dried under an airflow at 43% RH at room temperature. Seeds were subsequently hermetically stored at 4 °C for 20 months before phenotyping.

### 4.2. Seed Trait Phenotyping

To assess germination, germination speed and homogeneity, triplicates of 30 seeds were imbibed in 5 mL of deionised water on a filter paper (Whatman No1) in 9 cm diameter Petri dishes at 20 °C in the dark. Seeds with a protruding radicle length >2 mm were considered germinated. Germination were scored daily and final germination percentages were measured after 8 days of imbibition. To assess whether non germinated seeds were dormant, they were treated with 4 mL of 30 mM KNO_3_ solution, incubated for 5 days at 4 °C to allow the release of residual primary dormancy. Thereafter, they were transferred back to 20 °C in the dark and newly germinated seeds were counted eight days later. Germination capacity was calculated as the sum of the percentages of germination before and after the dormancy release treatment. Germination speed was assessed as the time required for the seed batch to reach 50% of germination (t50) from the fit of a three-parameter log-logistic model using R/drc package (version 3.0-1). Germination homogeneity (t80t20) was measured as the time difference between 20% and 80% of germination using the same fit. Both t50 and t80t20 were measured on the germination curve before dormancy release treatment.

To assess longevity, 2 replicates of 50 seeds were equilibrated in hermetically sealed box containing a saturated NaCl solution generating 75% RH at 20 °C for 7 days. They were transferred in laminated bags that were hermetically sealed and incubated at 35 °C for 110 days. Thereafter, seeds were retrieved and imbibed as described above. Thereafter, non germinated seeds were subjected to a 5 days cold stratification in the presence of 30 mM KNO_3_ before being incubated back to 20 °C in the dark for another 10 days. Survival corresponded to the sum of germination percentages before and after the dormancy release treatment.

### 4.3. Statistical Analysis of Phenotypic Traits and Heritability

Fixed effect of genotype (G), environment (E) and the genotype by environment interaction (G x E) per trait and condition was calculated by a two-way ANOVA in R/stats package, (version 3.5.0) (R Core Team, 2019) using the following linear model:

*Yij* = μ + *Gi* + *Ej* + G*Eij + *Ԑijk*, (1)

where *Yij* represents the phenotype of genotype *I* (*Gi*) and environment *j* (*Ej*), G*Eij the genotype by environment interaction and *Ԑijk* the residual effect. The total sum square was then partitioned in proportion and attributed to genotype, environment, genotype by environment or residual effect.

Broad-sense heritability (*h*2) was computed for each trait and condition by using the following linear model:

*Yi* = μ + *Gi* + *Ԑij* (2)

where *Gi* represents the random effect of genotype and *Ԑij* the random residual effect. Then, heritability was derived from the variance components of the model:

*h*2 = σ2 g/(σ2 g + σ2 e) (3)

where σ2 g and σ2 e are the genetic and residual variances, respectively.

Significant differences between control and heat stress condition for seed vigour traits for the 8 MAGIC parental lines were tested using a z-test (*p* < 0.05) for longevity and a *t*-test (*p* < 0.05) for germination, germination capacity, t50 and t80t20.

### 4.4. QTL Analyses

QTL analyses were performed on SL2.4 genome version using R/qtl2 package, version 0.20 [48] designed for QTL detection in multi-parental populations. Percentages of germination, germination capacity and survival after 110 days of storage were first transformed into probit, with values of 100% and 0% set at 99.9% and 0.1%, respectively. For the other phenotypic data, values were first normalized using the Box Cox function implemented in the R package MASS, version 7.3-51.5 [49]. Then for every trait, mean value per genotype and per condition or plasticity were used as phenotypic values for QTL mapping. The plasticity index was calculated as (Control–Stress)/Control. Genotype probabilities were first calculated using the calc.genoprob function. QTL mapping was executed using the linear mixed model, accounting for relationships among individuals using a random polygenic effect. The LOCO (leave one chromosome out) procedure, which scan each chromosome using a kinship matrix, which is calculated using data from all other chromosomes, was applied. MAGIC genotype data are available at https://doi.org/10.15454/UVZTAV (accessed on 2 November 2020). Significance thresholds for QTL detection were determined using a 5% Bonferroni threshold, leading to a LOD score threshold value of 4.43. Confidence intervals for each QTL were defined by dropping one unit of LOD score. The LOD score was dropped of two units to separate two significant peaks as distinct QTL.

### 4.5. Identification of Candidate Genes Located under the QTL

Candidate genes located under QTL with a confidence interval shorter than 3Mb were investigated on SL4.0 tomato genome by mapping marker positions from SL2.40 (ITAG2.3) to SL4.0. For each QTL, the number of genes and polymorphisms within the CI were identified based on the sequence information of all parental lines and the reference genome SL2.4 (Tomato Genome consortium, 2012 available at https://solgenomics.net/organism/Solanum_lycopersicum/genome (accessed on 3 November 2020). Identified genes and polymorphisms were first narrowed down based on the parental allelic effect at the QTL (Table S1). Then, candidate gene lists were filtered using Moneymaker gene expression kinetics from Bizouerne et al. [11,22] to select genes with transcript levels detected in tomato seed tissues.

## 5. Conclusions

This work highlights the importance of the environment during fruit development in determining seed vigour and longevity. There was a high genetic variability in the response to heat stress in tomato seed. We showed that using the seed phenotypic plasticity as a trait *per se* is a powerful approach for detecting heat-response QTLs associated with longevity and seed vigour. Several candidate genes were identified that were associated with DNA repair, cell wall, lipid polyester synthesis or represented regulatory hubs associated with ABA and ethylene response. This study also points out several *DOG1* and *DOG-like* genes whose function need to be deciphered. Altogether this work provides tomato genetic resources that will be helpful in unravelling the regulatory components governing heat stress tolerance in seeds and in developing varieties whose seeds are resilient to the effects of climate changes.

## Figures and Tables

**Table 1:** Phenotypic variation for seed vigour traits in control and heat stress conditions. h2 correspond to broad sense heritability. Germination capacity represents the sum of germination percentages obtained before and after dormancy release using stratification and KNO_3_ treatment.

Trait	Environment	Parent Range	MAGIC Lines			h^2^
			Min	Max	Mean	
Longevity (%)	Control	12–98	0	100	62	0.88
	Heat stress	0–98	0	98	32	0.88
Germination	Control	65–100	9	100	92	0.93
(%)	Heat stress	65–98	20	100	86	0.88
Germination capacity (%)	Control	76–100	41	100	95	0.86
	Heat stress	68–98	20	100	87	0.87
t50 (d)	Control	2.16–4.86	1.89	6.27	3.25	0.95
	Heat stress	2.65–4.24	2.09	9.19	3.51	0.85
t80t20 (d)	Control	0.32–1.08	0.16	2.31	0.7	0.72
	Heat stress	0.52–1.14	0.16	4.9	1.16	0.57

**Table 2:** Phenotypic variation attributed to the genotype (G), the environment (E) and the genotype by environment (G × E) interactions. *** = *p*-value < 0.001. Germ, germination. SSq, sum of squares.

Traits	G	SSq G %	E	SSq E %	GxE	SSq GxE %	SSq Resid %
Longevity (%)	<2 × 10^−16^ ***	64.41	<2 × 10^−16^ ***	12.98	<2 × 10^−16^ ***	17.66	4.95
Germination (%)	<2 × 10^−16^ ***	64.78	<2 × 10^−16^ ***	4.38	<2 × 10^−16^ ***	24.75	6.09
Germ. capacity (%)	<2 × 10^−16^ ***	55.60	<2 × 10^−16^ ***	9.11	<2 × 10^−16^ ***	27.13	8.16
t50 (d)	<2 × 10^−16^ ***	70.01	<2 × 10^−16^ ***	1.16	<2 × 10^−16^ ***	22.49	6.34
t80t20 (d)	<2 × 10^−16^ ***	52.01	<2 × 10^−16^ ***	7.73	<2 × 10^−16^ ***	19.43	20.82

**Table 3:** Characteristics of detected QTLs. ci, confidence interval; chr, chromosome; lo, low; hi, high; germ, germination.

Marker	Trait	Condition			QTL Position according to Genetic Distance				QTL Position according to Physical Distance			
			chr	LOD	pos	ci_lo	ci_hi	ci_cM	posMb	ci_loMb	ci_hiMb	ci_Mb
X06_44719903	germination	stress	6	4.6	135.9	117	141	24	44.5	41.4	46	4.60
X10_62014380	germination	stress	10	5.2	101.2	54.4	102.2	47.8	62	59.5	62.6	3.10
X11_504246	germination	plasticity	11	7.1	15	0	30.1	30.1	1.1	0	2	2.00
X06_33306736	germ capacity	control	6	4.9	20.4	12.5	30.4	17.9	33.4	31.9	33.7	1.80
X07_62159652	germ capacity	control	7	4.5	161.6	141.6	182.7	41.1	62.1	58.4	63.9	5.50
X11_7241787	germ capacity	control	11	5.5	97.6	88.1	101.7	13.6	5.5	5	26.9	21.90
X04_12083701	germ capacity	stress	4	6.1	61.8	57	86.4	29.4	17	5.3	56.5	51.20
X11_1987728	germ capacity	stress	11	4.7	30.1	19.8	167.5	147.7	2	1.4	53	51.60
X02_36220609	longevity	control	2	5.59	97.02	68.99	108.70	39.71	36.22	34.03	36.39	2.35
X02_39125317	longevity	control	2	4.71	135.22	124.29	159.14	34.85	39.74	38.74	44.26	5.52
X09_1759444	longevity	control	9	4.32	15.62	6.39	56.54	50.15	2.10	1.19	4.03	2.83
X01_78925532	longevity	stress	1	4.15	134.89	124.94	138.48	13.54	78.78	78.20	79.99	1.80
X02_45506212	longevity	stress	2	4.45	168.43	153.10	193.05	39.96	45.76	43.05	47.50	4.45
X09_1062246	longevity	stress	9	5.67	5.36	0.71	6.62	5.90	1.12	0.59	1.24	0.64
X02_44814312	longevity	plasticity	2	4.27	161.82	158.64	178.77	20.13	44.52	43.94	46.35	2.42
X05_4985019	longevity	plasticity	5	4.56	42.10	37.90	55.31	17.41	4.97	4.32	5.89	1.56
X06_39119433	longevity	plasticity	6	4.50	103.37	59.04	131.27	72.23	39.12	35.33	43.67	8.33
X07_51090892	longevity	plasticity	7	4.98	77.46	56.21	79.37	23.16	51.09	3.08	53.09	50.01
X11_5520602	longevity	plasticity	11	5.64	97.56	59.01	104.45	45.45	7.24	4.15	29.34	25.19
X12_64391888	longevity	plasticity	12	5.76	178.20	159.25	186.18	26.92	64.29	63.49	65.48	1.99
X02_35922016	t50.1	control	2	5.47	92.59	68.99	108.7	39.71	35.92	34.03	36.39	2.35
X02_43477320	t50.2	control	2	5.15	158.64	149.75	161.82	12.07	43.84	42.4	44.52	2.12
X04_60240094	t50	control	4	5.29	138.73	132.88	151.95	19.07	60.24	59.6	60.96	1.37
X07_2671204	t50	control	7	4.43	47.53	20.04	56.21	36.17	2.67	0.85	3.08	2.23
X09_61268966	t50	control	9	4.67	113.9	79.09	162.3	83.21	61.27	4.55	64.61	60.06
X11_50118788	t50	control	11	5.05	130.7	119.79	141.67	21.88	50.12	49.51	50.91	1.4
X12_8722237	t50	control	12	6.4	69.06	54.89	85.34	30.45	5.27	4.19	46.23	42.05
X01_78197981	t50	stress	1	5.01	124.94	110.81	135.54	24.73	78.18	77.33	79.3	1.97
X02_35852446	t50	stress	2	4.65	88.66	68.99	159.14	90.15	35.85	34.03	44.26	10.22
X04_62201077	t50	stress	4	5.11	178.84	138.73	192.21	53.49	62.13	60.24	62.83	2.59
X09_30052623	t50	stress	9	6.04	98.14	88.03	108.25	20.23	23.38	8.33	59.31	50.98
X10_2344795	t50	stress	10	4.97	27.96	20.36	35.15	14.79	2.34	2.22	3.59	1.37
X04_59595681	t50.1	plasticity	4	5.38	132.88	119.51	142.51	22.99	59.6	58.87	60.43	1.56
X04_62339583	t50.2	plasticity	4	5.7	180.94	175.38	192.21	16.83	62.23	62.12	62.83	0.71
X11_2573606	t50	plasticity	11	4.89	38.93	0	141.67	141.67	2.57	0.01	50.91	50.9
X02_44746073	t80t20	control	2	4.86	161.82	158.64	165.73	7.09	44.52	43.94	44.89	0.95
X03_60247504	t80t20	control	3	4.99	189.68	172.43	198.13	25.7	60.25	58.5	60.98	2.47
X12_5047381	t80t20	control	12	4.47	67.99	45.52	81.58	36.06	5.05	3.67	44.68	41.01
X08_57563157	t80t20	plasticity	8	4.58	79.49	67.22	100.91	33.69	57.87	56.71	59.97	3.26

**Table 4:** Number of genes and polymorphisms of the QTLs before and after filtering using the parental allelic effects detected in control or stressed growth conditions and exhibiting a confidence interval (CI) smaller than 3Mb. CG, candidate genes; CP candidate polymorphism; Cer, Cervil; Crio, Criollo; Fer, Ferum; LA0, LA0147; LA14, LA1420; Lev, Levovil; Plov, Plovdiv; Stup, Stupicke. Filtered nb CG, retained CG after filtering for contrasting allelic effect of parents; seed exp CG, number of filtered CG with transcripts present in seed tissues. C, control; S, heat stress conditions; GermC, germination capacity. Parental effects and filters are presented in Table S1.

Marker	Trait	Chr	ci_Mb	Nb Gene	Nb pol	Filtered nb CG	Filtered nb CP	Seed exp CG	Seed exp CP
X01_78197981	t50_S	1	1.97	238	24,566	238	10,979	174	8496
X01_78925532	longevity_S	1	1.97	213	20,457	141	687	108	570
X02_35922016	t50.1_C	2	2.35	278	22,018	277	16,611	222	13,788
X02_36220609	longevity_C	2	2.35	293	37,679	231	3033	185	2542
X02_43477320	t50.2_C	2	2.12	278	18,239	228	2427	197	2095
X02_44746073	t80t20_C	2	0.95	141	7106	123	1339	76	190
X02_44814312	longevity_P	2	2.42	354	31,866	236	1596	199	1374
X03_60247504	t80t20_C	3	2.47	332	23,667	284	2698	230	2274
X04_59595681	t50.1_P	4	1.56	145	20,401	73	219	65	208
X04_60240094	t50_C	4	1.37	151	18,014	10	16	6	12
X04_62201077	t50_S	4	2.59	333	24,049	23	36	17	28
X04_62339583	t50.2_P	4	0.71	97	4402	18	32	14	27
X05_4985019	longevity_P	5	1.56	162	30,192	155	4608	117	3581
X06_33306736	germC_C	6	1.8	132	27,051	98	451	76	354
X07_2671204	t50_C	7	2.23	218	14,125	176	1070	147	813
X09_1062246	longevity_S	9	0.64	88	8951	30	97	28	85
X09_1759444	longevity_C	9	2.83	308	45,197	226	6398	181	5033
X10_2344795	t50_S	10	1.37	121	2257	76	498	54	270
X11_50118788	t50_C	11	1.4	156	10,643	24	123	20	115
X11_504246	germination_P	11	2	283	23,523	203	1406	166	1180
X12_64391888	longevity_P	12	1.99	332	22,890	241	1434	173	1025

## Data Availability

The original data that are not presented in this study are available on request from the corresponding author. The reference tomato genome SL2.4 used in this study is available at https://solgenomics.net/organism/Solanum_lycopersicum/genome (accessed on 3 November 2020) (Tomato Genome consortium, 2012). MAGIC genotype data used this study are available at https://doi.org/10.15454/UVZTAV (accessed on 2 November 2020). RNAseq data used in this study are from the NCBI Gene Expression Omnibus repository, (GSE155838) available at https://www.ncbi.nlm.nih.gov/geo/query/acc.cgi?acc=GSE155838 (accessed on 20 September 2023).

## Supplementary Materials

The following supporting information can be downloaded at: https://www.mdpi.com/article/10.3390/plants12203632/s1, Table S1. Parental allelic effects on indicated QTLs detected in control or stressed growth conditions exhibiting a confidence interval (CI) smaller than 3Mb together with number of genes and polymorphisms of the QTL before and after filtering. Table S2. Genes underlying QTL regions of seed vigour traits. Table S3. Polymorphisms of candidate genes. Figure S1. Correlation matrix for measured seed traits in control (C) and heat stress (S) growth condition.
