# Supplementary material for: Genetic Variability in Seed Longevity and Germination Traits in a Tomato MAGIC Population in Contrasting Environments
Source: Plants (Basel). 2023 Oct 20;12(20):3632. doi: 10.3390/plants12203632 (PMC10610530; doi:10.3390/plants12203632)
Supplement: Supplementary file 1 [file plants-12-03632-s001.zip › Figures S1 _Leprince.pptx]

## Slide 1
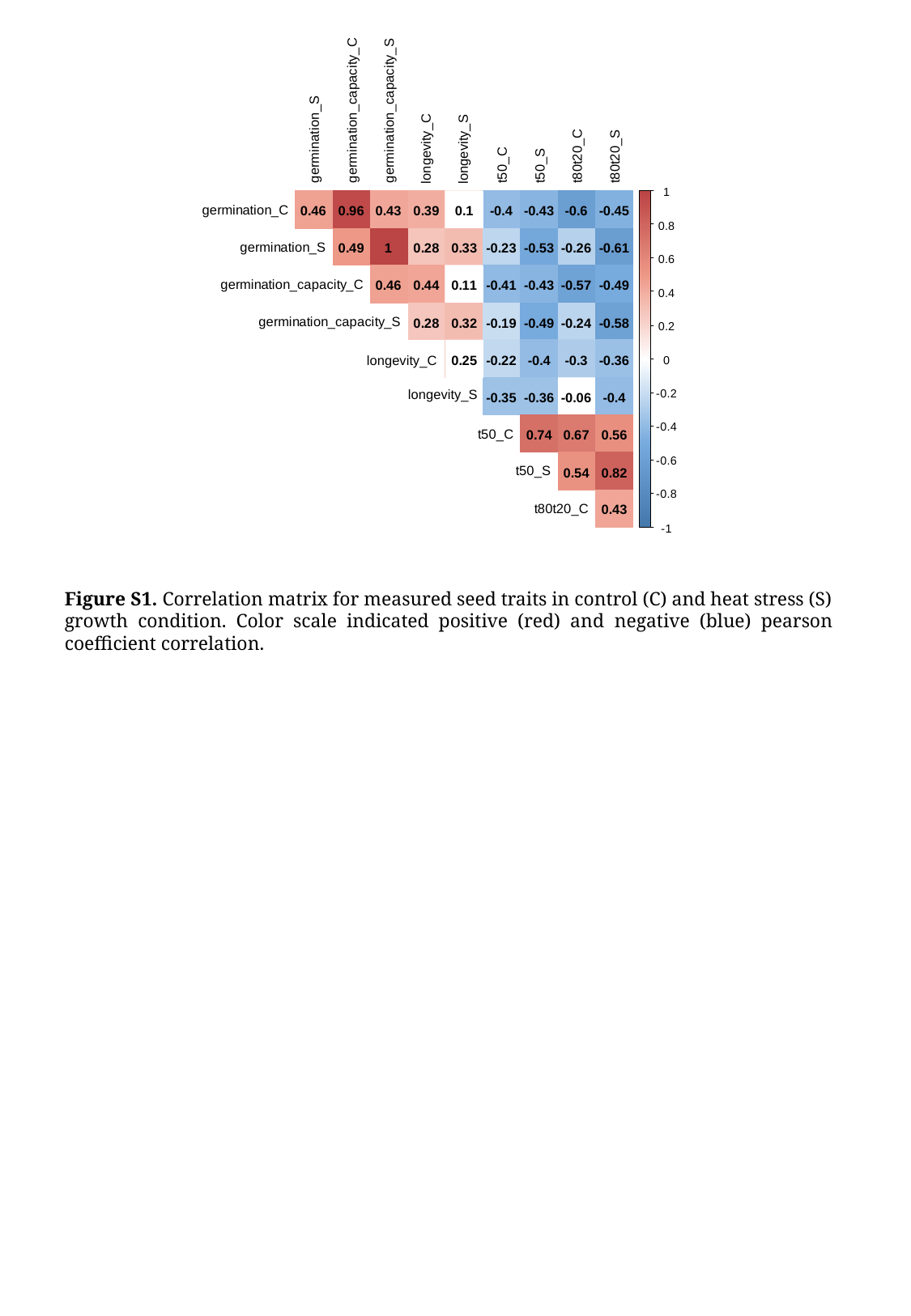

longevity_S
longevity_C
longevity_C
longevity_S
Figure S1. Correlation matrix for measured seed traits in control (C) and heat stress (S) growth condition. Color scale indicated positive (red) and negative (blue) pearson coefficient correlation.
